# Antivirals for adult patients hospitalized with SARS-CoV-2 infection: A randomized, Phase II/III, multicenter, placebo-controlled, adaptive study, with multiple arms and stages. COALITION COVID-19 BRAZIL IX – REVOLUTIOn: protocol and statistical analysis plan

**DOI:** 10.5935/0103-507X.20220002-en

**Published:** 2022

**Authors:** Israel Silva Maia, Aline Marcadenti, Fernando Godinho Zampieri, Lucas Petri Damiani, Renato Hideo Nakagawa Santos, Karina Leal Negrelli, Samara Pinheiro do Carmo Gomes, Jaqueline Oliveira Gomes, Mariana Barbosa dos Santos Carollo, Tamiris Abait Miranda, Eliana Santucci, Nanci Valeis, Ligia Nasi Laranjeira, Glauco Adrieno Westphal, Jacques Gabriel Alvares Horta, Uri Adrian Prync Flato, Camilo Fernandes, Waldemar Carlos Barros, Renata S Bolan, Otávio Celso Eluf Gebara, Meton Soares de Alencar Filho, Victor Augusto Hamamoto, Mauro Esteves Hernandes, Nicole Alberti Golin, Ronald Torres de Olinda, Flávia Ribeiro Machado, Régis Goulart Rosa, Viviane Cordeiro Veiga, Luciano César Pontes de Azevedo, Alvaro Avezum, Renato Delascio Lopes, Tiago Moreno L Souza, Otávio Berwanger, Alexandre Biasi Cavalcanti

**Affiliations:** 1 Research Institute, HCor-Hospital do Coração - São Paulo (SP), Brazil.; 2 Division of Anesthesiology, Faculdade de Medicina, Universidade de São Paulo - São Paulo (SP), Brazil.; 3 Brazilian Research in Intensive Care Network (BRICNet) - São Paulo (SP), Brazil.; 4 Centro Hospital Unimed Joinvile - Joinvile (SC), Brazil.; 5 Santa Casa de Misericórdia de Ouro Preto - Ouro Preto (MG), Brazil.; 6 Universidade de Marilia - Marilia (SP), Brazil.; 7 Hospital Nereu Ramos - Florianópolis (SC), Brazil.; 8 Unimed Sul Capixaba - Cachoeiro de Itapemirim (ES), Brazil.; 9 Hospital Baia Sul - Florianópolis (SC), Brazil; 10 Hospital Santa Paula - São Paulo (SP), Brazil.; 11 Hospital São Vicente de Paula - Barbalha (CE), Brazil.; 12 International Research Center, Hospital Alemão Oswaldo Cruz - São Paulo (SP), Brazil.; 13 Santa Casa de Votuporanga - Votuporanga (SP), Brazil.; 14 Hospital Tachini - Bento Gonçalves (RS), Brazil.; 15 Hospital São Francisco - Brasília (DF), Brazil.; 16 Department of Anaesthesiology, Pain and Intensive Care Medicine, Universidade Federal de São Paulo (SP), Brazil.; 17 Hospital Moinhos de Vento - Porto Alegre (RS), Brazil.; 18 BP - A Beneficência Portuguesa de São Paulo - São Paulo (SP), Brazil.; 19 Research and Education Institute, Hospital Sírio-Libanês - São Paulo (SP), Brazil.; 20 Brazilian Clinical Research Institute (BCRI) - São Paulo (SP), Brazil.; 21 Duke University Medical Center, Duke Clinical Research Institute - Durham, North Carolina, United States.; 22 Laboratório de Imunofarmacologia, Instituto Oswaldo Cruz, Fundação Oswaldo Cruz - Rio de Janeiro (RJ), Brazil.; 23 National Institute for Science and Technology on Innovation in Diseases of Neglected Populations, Center for Technological Development in Health, Fundação Oswaldo Cruz - Rio de Janeiro (RJ), Brazil.; 24 Hospital Israelita Albert Einstein - São Paulo (SP), Brazil.

**Keywords:** COVID-19, Coronavirus infections, Antiviral agents, Protocol, Respiratory insufficiency, Daclatasvir, Sofosbuvir

## Abstract

Repurposed drugs are important in resource-limited settings because the interventions are more rapidly available, have already been tested safely in other populations and are inexpensive. Repurposed drugs are an effective solution, especially for emerging diseases such as COVID-19. The REVOLUTIOn trial has the objective of evaluating three repurposed antiviral drugs, atazanavir, daclatasvir and sofosbuvir, already used for HIV- and hepatitis C virus-infected patients in a randomized, placebo-controlled, adaptive, multiarm, multistage study. The drugs will be tested simultaneously in a Phase II trial to first identify whether any of these drugs alone or in combination reduce the viral load. If they do, a Phase III trial will be initiated to investigate if these medications are capable of increasing the number of days free respiratory support. Participants must be hospitalized adults aged ≥ 18 years with initiation of symptoms ≤ 9 days and SpO_2_ ≤ 94% in room air or a need for supplemental oxygen to maintain an SpO_2_ > 94%. The expected total sample size ranges from 252 to 1,005 participants, depending on the number of stages that will be completed in the study. Hence, the protocol is described here in detail together with the statistical analysis plan. In conclusion, the REVOLUTIOn trial is designed to provide evidence on whether atazanavir, daclatasvir or sofosbuvir decrease the SARS-CoV-2 load in patients with COVID-19 and increase the number of days patients are free of respiratory support. In this protocol paper, we describe the rationale, design, and status of the trial.

**ClinicalTrials.gov identifier:** NCT04468087

## INTRODUCTION

### Background and rationale

Coronavirus disease 2019 (COVID-19) reached a pandemic status, and several approaches have been suggested to control severe acute respiratory syndrome coronavirus 2 (SARS-CoV-2) replication in patients with moderate to severe cases. One of these strategies includes the repurposing of antiviral drugs used to treat severe acute respiratory syndrome coronavirus 1 (SARS-CoV-1) and Middle East respiratory syndrome coronavirus (MERS-CoV), including antiretroviral agents.^(1)^ Drugs such as remdesivir, lopinavir and ritonavir, darunavir and cobicistat, umifenovir and oseltamivir potentially exert therapeutic effects against COVID-19.^(2)^ However, among these repurposed drugs, only remdesivir showed clinical benefits for COVID-19 treatment.^(3)^

Other specific and nonspecific antivirals have been proposed to treat the disease. Some are based on the results from in vitro and clinical studies conducted with a small sample size in specific populations. Atazanavir (ATV) blocked the major protease activity of SARS-CoV-2 in a protease-free cell assay;^(4)^ sofosbuvir (SOF) showed EC_50_ values against SARS-CoV-2 replication of 6.2 and 9.5µM in HuH7 (hepatoma) and Calu-3 (type II pneumocytes) cells, respectively;^(5)^ and daclatasvir (DCV) consistently inhibited the production of SARS-CoV-2 infectious particles in Vero cells, the HuH-7-cell line and Calu-3 cells, preventing the induction of interleukin (IL) 6 and tumor necrosis factor alpha (TNF-α) production.^(5)^ Available clinical studies are synthesized in recent meta-analysis, SOF/DCV may reduce the mortality rate and need for intensive care unit (ICU)/invasive mechanical ventilation (IMV) in patients with COVID-19 while increasing the chance for clinical recovery with a low to moderate quality of evidence.^(6)^ However, no studies investigating DCV or ATV alone have been published.

Implementing studies that allow more than one new treatment to be tested simultaneously may be advantageous over classic parallel group studies. The main objectives of this type of clinical trial are to quickly reject any new therapies that do not seem to be better than controls and to identify those that are significantly better in terms of clinical outcomes.^(7)^ Thus, we propose a randomized, placebo (PbO)-controlled, adaptive, multiarm, multistage Phase II trial to evaluate ATV, DCV and DCV plus SOF simultaneously to first identify whether any of these drugs alone or in combination reduce the viral load. If they do, a Phase III trial will be initiated to investigate clinical outcomes.

### Objectives

The main objective of the REVOLUTIOn trial is to evaluate whether repurposed antiviral drugs alone or in combination are effective at decreasing the viral load and increasing the number of days free of respiratory support. The primary and secondary objectives are described in table 1.

**Table 1:** Objectives and outcomes

Primary objective	Outcome/primary variables
Phase II first stage (II/1): compare the effect of treatment with single antivirals against placebo in reducing the load of SARS-CoV-2 in nasopharyngeal swab samples	Decay rate (slope) of the SARS-CoV-2 viral load logarithm in nasopharyngeal and swab samples evaluated at D0, D3, D6 and D10 after randomization
Phase II second stage (II/2): compare the effect of treatment with combinations of antivirals compared to isolated ones in reducing the SARS-CoV-2 viral load in nasopharyngeal swab samples	Decay rate (slope) of the SARS-CoV-2 viral load logarithm in nasopharyngeal and swab samples evaluated at D0, D3, D6 and D10 after randomization
Phase III: compare the efficacy of antivirals alone or in combination to the placebo in increasing the number of days free of respiratory support	Days free of respiratory support, defined as the number of days without oxygen, noninvasive ventilation/high-flow nasal cannula or the need for mechanical ventilation within 15 days from randomization 1. This parameter is counted as follows: D = zero (if the patient dies within 15 days (either in the hospital or at home or remains on respiratory support with oxygen through a nasal catheter, noninvasive ventilation, high-flow nasal catheter, or mechanical ventilation ≥ 15 days) D = 15 - x (if the patient is released from the hospital in < 15 days, where x represents the number of days with respiratory support during hospitalization)
Secondary objectives	Outcomes/secondary variables
Evaluate the status using the 7-stage ordinal scale for clinical outcomes on D15	Percentage of patients in various stages: 1. Not hospitalized with resumption of normal activities 2. Not hospitalized, but unable to resume normal activities 3. Hospitalized, with no need for supplemental oxygen 4. Hospitalized, requiring supplemental oxygen 5. Hospitalized, requiring high-flow nasal oxygen therapy, noninvasive mechanical ventilation, or both 6. Hospitalized, requiring blood oxygenation through a membrane system, invasive mechanical ventilation or both 7. Death
Evaluate the status using the 6-stage ordinal scale for clinical outcomes on D7	Percentage of patients in various stages: 1. Nonhospitalized 2. Hospitalized, with no need for supplemental oxygen 3. Hospitalized, requiring supplemental oxygen 4. Hospitalized, requiring high-flow nasal oxygen therapy, noninvasive mechanical ventilation, or both 5. Hospitalized, requiring blood oxygenation through a membrane system, invasive mechanical ventilation or both 6. Death
Evaluate 28-day mortality	Percentage of deaths in 28 days
Evaluate the number of days free from mechanical ventilation within 28 days	D = 28 - number of days requiring mechanical ventilation D = zero if death occurs or the patient continues to require mechanical ventilation after 28 days
Evaluate the number of days out of the hospital within 28 days	D = 28 - number of days after admission to the hospital D = zero if death occurs or the patient remains hospitalized after 28 days
Evaluate the time to discharge	Number of days from randomization to discharge, within 28 days D = 28 - number of days from randomization to hospital discharge D = zero if death occurs or the patient remains hospitalized after 28 days
Evaluate the number of days free of respiratory support within 15 days for Phases II/1 and II/2	D = 15 - number of days with respiratory support on hospitalization D = zero if death occurs or the patient remains hospitalized with a need for respiratory support defined as the use of low-flow, high-flow oxygen, IMV, or MV in 15 days
Safety objective	Outcomes/safety variables
Evaluate Grade 2, 3 or 4 adverse events, which were not present at the patient’s entrance, defined by the Division of AIDS table for Grading the Severity of Adult and Pediatric Adverse Events^(8)^	Percentage of Grade 2, 3, or 4 adverse events in the Division of AIDS table
Evaluate serious adverse events Percentage of serious adverse events	Evaluate discontinuation of study drug-related treatment Percentage of patients who needed to discontinue the intervention (study drug)

D - day; IMV - invasive mechanical ventilation; MV - mechanical ventilation.

### Study design

**Figure 1:**
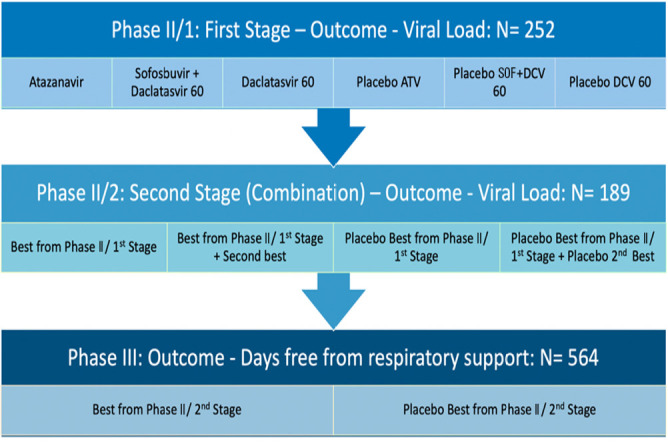
Study flowchart. ATV - atazanavir; SOF - sofosbuvir; DCV - daclatasvir.

A randomized, adaptive, PbO-controlled, multiarm, multistage trial conducted in 3 continuous stages (ClinicalTrials.gov Identifier: NCT04468087); version 4.0 of Protocol 10/07/2020. The first two stages are Phase II studies, and the third is a Phase III study, as shown in figure 1.

## METHODS

### Study settings

The study will be conducted in approximately 60 Brazilian hospitals.

### Eligibility criteria

The inclusion and exclusion criteria are described in table 2. Procedures for the initial diagnosis of COVID-19, collection of biological material, viral identification, genetic sequencing and viral isolation in culture are explained in Supplementary material 1.

**Table 2:** Inclusion and exclusion criteria

Inclusion criteria	Exclusion criteria
1. Adults (≥ 18 years) hospitalized with COVID-19 with one of the following conditions: - Positive RT-PCR test for SARS-CoV-2 OR - Typical clinical history AND chest CT with typical findings, pending the results of the RT-PCR test for SARS-CoV-2 2. Time between symptom onset and inclusion ≤ 9 days 3. SpO2 ≤ 94% in room air or need for supplemental oxygen to maintain an SpO2 > 94% 4. The patient consents to participate in the study and is willing to comply with all study procedures, including the collection of virology samples	Presence of any of the following conditions: - Patients requiring invasive mechanical ventilation - ALT or AST level > 5 times the upper limit of normal - Total bilirubin level > 2mg/dL - Platelet count < 50,000 cells/L - Total neutrophil count < 750 cells/L - Renal failure (eGFR < 30mL/min/1.73m^^2^^, using the MDRD or CKD-EPI method); and predefined renal failure Stage 3 according to the AKIN^(9)^ classification with a serum creatinine level > 4mg/dL or patient already on renal replacement therapy - History of liver disease with moderate to severe impairment (liver cirrhosis with - Child Pugh B and C classification) previously known* - Decompensated congestive heart failure* - Pregnant or breastfeeding patients - Known allergy or hypersensitivity to any study drug - Carrier of hepatitis C (positive HCV RNA), active hepatitis B (positive surface antigen in the past), or HIV (ELISA and confirmatory Western blot in the past) - Patients currently using nucleoside or nucleotide analog drugs for any indication - Corrected QT interval > 480 on the electrocardiogram - Heart rate < 55 bpm - Patients using or who recently used (< 90 days) amiodarone - Women of childbearing potential* or men with a partner of childbearing potential who do NOT agree to use two contraceptive methods (including barrier method) for 100 days

RT-PCR - real time polymerase chain reaction; CT - computed tomography; SpO2 - oxygen saturation; ALT - alanine aminotransferase; AST - aspartate aminotransferase; eGFR - estimated glomerular filtration rate; MDRD - Modification of Diet in Renal Disease; CKD-EPI - Chronic Kidney Disease Epidemiology Collaboration; AKIN - Acute Kidney Injury Network; HCV - hepatitis C virus; ELISA - enzyme-linked immunosorbent assay.

* Defined in Supplementary material 1.

#### Interventions

Intervention drugs administered in each stage

**Phase II, first stage -** six arms with six different interventions will be designed:

1. ATV.
2. DCV.
3. SOF/DCV.
4. PbO ATV.
5. PbO DCV.
6. PbO SOF/DCV.

**Phase II, second stage -** four arms with four different interventions will be designed:

1. Best of the first stage.
2. Best of the first stage + the second best.
3. PbO best first stage.
4. PbO best first stage + PbO second best.

**Phase III, third stage -** two arms with two different interventions will be designed:

1. Best antiviral alone or in combination (defined in Stage 1 or 2).
2. PbO best antiviral alone or in combination.

The doses and administration routes of the study drugs are described in table 3. If swallowing is impossible for any reason, the protocol provides for the use of the study drugs via a nasogastric or nasoenteral tube. The study drugs will be donated by pharmaceutical companies, with ATV (and PbO) donated by Fundação Oswaldo Cruz (Fiocruz) and DCV, SOF and respective PbOs donated by Blanver Farmoquímica e Farmacêutica.

**Table 3:** Study drug dosage

Drug	Pharmaceutical form	Route of administration/ instructions for use	Frequency of administration	Conservation
Daclatasvir/placebo DCV	Coated tablets/ 28 tablets bottles*	Orally with or without food	2 tablets once daily on D1, 1 tablet once daily from D2 to D10	Room temperature 15 to 30°C
Sofosbuvir/placebo SOF	Coated tablets/ 28 tablets bottles*	Orally with or without food	1 tablet twice daily on D1, 1 tablet once daily from D2 to d10	Room temperature 15 to 30°C
Atazanavir/placebo ATV	Capsules with packs of 30 capsules*	Orally with food	2 capsules twice daily on D1, 1 capsule twice daily from D2 to d10	Room temperature 15 to 30°C

DCV - daclatasvir; SOF - sofosbuvir; ATV - atazanavir; D - day.

* Coated tablets for daclatasvir and sofosbuvir treatments and placebos and capsules for atazanavir treatment and placebo are identical in physical appearance.

#### Discontinuation of the drug treatment and safety criteria

The set of primary analyses for safety and efficacy will include individuals who received at least one dose of the study drug. Emerging treatment data will be analyzed and defined as data collected from the administration of the first study drug dose to the date of administration of the last study drug dose plus 30 days.

At medical discretion, the study drug(s) will be discontinued if a research participant meets one of the following criteria:

1. The researcher considers that the discontinuation of the study drug is in the best interest of the research participant.
2. Elevated levels of alanine aminotransferase (ALT) or aspartate aminotransferase (AST) greater than ten times the value of the upper limit of normal (ULN). Elevations in ALT or AST levels often follow the clinical course of COVID-19, whether they are due to shock, sepsis or even direct SARS-CoV-2 infection in liver cells.^(10)^ These changes may occur in the two study groups: active drug or PbO.
3. Elevated levels of ALT or AST greater than 3x the ULN **WITHOUT** an increase in alkaline phosphatase levels confirmed in a new test performed within 48 hours **AND** the presence of one of the following conditions:
  - Total bilirubin level > 2 times the ULN.
  - International Normalized Ratio (INR) > 2.
4. Elevated ALT or AST levels greater than 3x the ULN accompanied by the presence of the following two conditions:
  - Onset or worsening of fatigue, nausea, vomiting, discomfort in the upper abdomen, or fever.
  - Rash and/or peripheral eosinophilia (> 5%).
5. Any Grade 3 or greater rash accompanied by symptoms.
6. Any Grade 4 AE or laboratory abnormality considered related to the study drug.

#### Discontinuation of one arm of the study for safety

The criteria for discontinuing one arm of the study for safety are the occurrence of one of the following 3 conditions:

1. Presence of the components of Hy’s Law^(11)^* which consists of the presence of all of the following conditions confirmed by an adjudicating committee of 2 independent evaluators:
  - The study drug causes a higher incidence of hepatocellular injury confirmed by a > three times ULN increase in ALT and AST levels than the PbO group.
  - Increase in ALT or AST levels > three times ULN and elevation of total bilirubin levels > two times ULN without signs of cholestasis (elevated alkaline phosphatase levels).
  - No other explanation for the combination of the changes described above, such as the presence of hepatitis A, B, C, use of vasopressors or IMV; acute or preexisting hepatic disease or the presence of another drug capable of causing the observed injury.
2. Elevated ALT and AST levels > five, ten or twenty times ULN more frequently in the treated group than in the control group after 48-hour reassessments.
3. Presence of more serious adverse events (SAEs) defined in Section S.5 (Supplementary material 1) in the treatment arm than in the PbO arm.

Patients with COVID-19 may progress to sepsisassociated liver injury, which makes characterizing all the Hy Law criteria in these patients very challenging. For this study, we will consider a modified definition of Hy’s Law that will be present when the 3 criteria described above are met in the absence of shock or severe acute respiratory failure with the need for mechanical ventilation.

#### Rules for changing the study stage (Figure 2)

**Figure 2:**
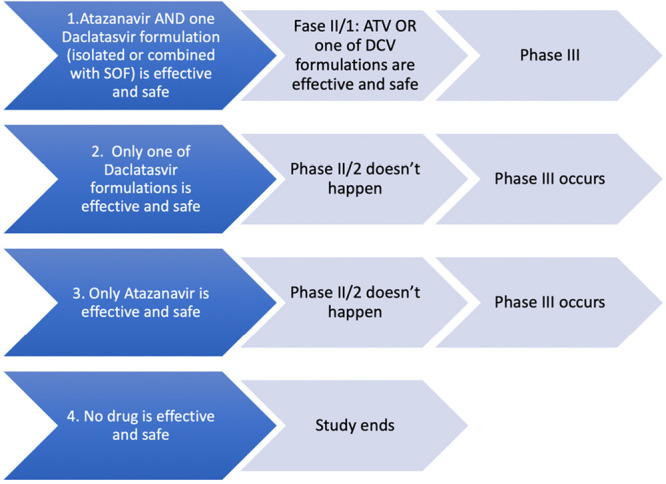
Rules for changing stages. SOF - sofosbuvir; ATV - atazanavir; DCV - daclatasvir.

1. If ATV and a presentation of DCV alone are effective and safe in the first stage, a second stage will be performed with the first- and second-best drugs from the previous stage combined according to the rate of decay of the viral load logarithm (slope). The drug with the highest decay rate will be administered first, followed by the drug with the second highest decay rate. The third phase will investigate the drug with the highest decay rate in Phase II/1 or Phase II/2.
2. If only DCV presentations are effective and safe, a Phase II/2 trial will not be conducted, and Phase III will start with the formulation of DCV alone or combined with SOF which produces the higher rate of decay and it is safe according to the interpretation of the DSMB.
3. If only ATV is effective and safe, a Phase II/2 will not be conducted, and Phase III starts with ATV alone.
4. If none of the study drugs are effective and safe in Phase II/1, the study ends without conducting Phase II/2 and/or Phase III.

#### All participants’ daily routines will include

1. A clinical evaluation by the attending physician.
2. Routine laboratory tests at the discretion of the attending physicians.
3. Respiratory and motor physiotherapy.
4. Surveillance of vital signs.
5. Addition of ventilatory support measures, such as increased oxygen flow, use of noninvasive ventilation or high-flow nasal cannula, as recommended by the attending physicians.
6. Prophylaxis of stress ulcers and venous thromboembolism according to the protocol of each institution.
7. Transfer to advanced units (ward and ICU) according to the clinical judgment and patient’s need.
8. The addition of other therapies, such as antibiotics, corticosteroids, IL-6 receptor inhibitors and other antivirals, as indicated by the attending physician.

### Study intervention compliance

- **Hospitalized patients:** Each dose of the study drug will be administered and supervised by a member of the clinical research team who is appropriately trained. The administration date and time will be entered into the case report form (CRF).
- **Out-of-hospital patients:** Patients who are discharged before the end of treatment will have their drug dispensed for home use and be monitored by telephone interviews, a medication diary and physical reevaluation in return visits on the missing day from the 4 prespecified schedule times (baseline and Days - D - 3, 6 and 10). Assessments of study medication use will be performed at each study visit. The subject should be instructed to bring all unused study drugs to each visit and any empty bottles. The dates and number of tablets dispensed and returned must be recorded on the drug accountability form.

#### Drug accountability

The principal investigator at each site may delegate responsibility for study product accountability to the research pharmacist at the participating site. He/she will be responsible for maintaining complete records and documentation of study product receipt, accountability, dispensation, storage conditions, and final disposition of the study product(s). The time of study drug administration to the subject will be recorded on the appropriate data collection form (CRF). All study product(s), whether administered or not, must be documented on the appropriate study product accountability record or dispensing log available at the pharmacy of each center. The Coordinating Center team will verify the study product accountability records and dispensing logs of each participating site.

#### Concomitant therapy and drug interaction

Therapy administered before enrollment with any other experimental treatment or off-label use of marketed medications must be discontinued upon enrollment. Concomitant therapy will be recorded daily until D10. A subject cannot participate in another clinical trial or receive any experimental treatment other than the study drugs for the treatment of COVID-19. Some concomitant medications must be stopped or adjusted according to table 4 due to drug interactions.

**Table 4:** Drug interactions

Drug	SOF	DCV	ATV	Effects	Action
Amiodarone	X	X	X	Bradycardia when administered with another antiviral	Do not administer. Switch to propafenone
Digoxin		X		↑Digoxin	Avoid administration. If necessary, decrease the dose by 30%
Quinidine		X	X	↑Quinidine	A dose adjustment is not needed (when not using ritonavir). Avoid use with DCV
Lidocaine			X		A dose adjustment is not needed (when not using ritonavir)
Carbamazepine	X	X	X	↓Efficacy SOF ↑Carbamazepine	Switch to 500mg of levetiracetam twice a day IV or VO
Phenytoin Phenobarbital				↓Efficacy ATV ↓Phenytoin ↓Phenobarbital	
Lamotrigine			X	↓Lamotrigine if administered with ritonavir	A dose adjustment is not needed (when not using ritonavir)
Rifampicin	X	X	X	↓Efficacy SOF and DCV	Do not administer or suspend RMP for 10 days
Itraconazole Ketoconazole Voriconazole		X X X	X X X	↑DCV ↑ATV ↑Itraconazole ↑Ketoconazole	Switch to amphotericin B
Dexamethasone		X		↓Efficacy DCV	Do not adjust dose. All patients will use this drug
Bosentan		X	X	↓Efficacy DCV	Switch to sildenafil at a dose of 25mg every 2 days
Dabigatran Rivaroxaban Apixaban		X	X	↑Dabigatran ↑Rivaroxaban ↑Apixaban	Substitute for enoxaparin
Warfarin	X	X	X	↑Warfarin	Monitor INR for a warfarin dose adjustment or switch to enoxaparin/heparin
Atorvastatin Pravastatin Simvastatin		X	X	↑Atorvastatin ↑Pravastatin ↑Simvastatin	Temporary suspension: risk of myopathy or statin dose reduction by 50%
Quetiapine			X	↑Quetiapine	Decrease quetiapine dose by 50%
Midazolam			X	↑Midazolam	Decrease midazolam dose by 50%
Ergot derivatives			X	↑Ergot derivatives	Suspend for 10 days
Omeprazole			X	↓Efficacy ATV	Switch to H2 antagonists. In case of gastrointestinal bleeding, maintain with the administration of the omeprazole dose with a difference of 12 hours from the ATV dose
Salmeterol			X	↑Salmeterol	Switch to formoterol or salbutamol
Sildenafil			X	↑Sildenafil	Reduce the dose of sildenafil to 25mg every 2 days
Tadalafil			X	↑Tadalafil	Reduce the tadalafil dose to 10mg every 72 hours
Antacids			X	↓Efficacy ATV	Administer 2 hours before or 1 hour after ATV
Tricyclic antidepressants Trazodone			X	↑Antidepressants ↑Trazodone	Decrease antidepressant doses by 50% Decrease the trazodone dose by 50%
Colchicine		X	X	↑Colchicine	Use a 0.6mg (1 capsule) dose followed by 0.3mg (1/2 tablet) 1 hour later. Do not readminister in less than 3 days
Diltiazem Nifedipine Verapamil		X	X	↑Diltiazem and DCV ↑Nifedipine and DCV ↑Verapamil and DCV	Reduce the diltiazem dose by 50% Reduce the nifedipine dose by 50% Reduce the verapamil dose by 50%
Famotidine Ranitidine Cimetidine			X	↓Efficacy ATV	Administer ATV 10 hours after and at least 2 hours before the famotidine dose Administer ATV 10 hours after and at least 2 hours before ranitidine dose Administer ATV 10 hours after and at least 2 hours before the cimetidine dose
Cyclosporine Tacrolimus Sirolimus			X	↑Cyclosporine ↑Tacrolimus ↑Sirolimus	Monitor serum level
Clarithromycin		X	X	↑DCV ↑ATV ↑Clarithromycin	Reduce the dose of clarithromycin by 50% (QTc prolongation) or use azithromycin
Oral contraceptives* containing ethinyl estradiol, norgestimate or norethindrone			X	↑Ethinyl estradiol ↑Norgestimate ↑Norethindrone	Exchange for desogestrel
Atazanavir		X		↑DCV only if using ritonavir	Do not change the dose Ritonavir will not be used
Fluticasone			X	↑Fluticasone	Switch to budesonide

DCV - daclatasvir; SOF - sofosbuvir; ATV - atazanavir; IV - intravenous; VO - oral; RMP - rifampicine; INR - International Normalized Ratio.

* Contraceptives: for patients in the sofosbuvir and daclatasvir groups, contraceptives with ethinyl estradiol will be provided. Patients participating in the atazanavir group will be provided contraceptives with desogestrel or gestodene.

### Outcomes

Primary, secondary and safety outcomes are described and defined in table 1.

### Sample size

We intend to include 252 patients in Phase II/Stage 1 (189 in the active groups and 63 in the PbO group) and 189 patients in Phase II/Stage 2 (126 in the active groups and 63 in the PbO group). For Phase III, 564 additional participants will be randomized (376 in the active group and 188 in the PbO group). Thus, at the end of Phase III, 314 patients will have been evaluated in the PbO group and between 439 and 502 patients will have been evaluated in the treatment group if the study persists through the three phases. More details about the sample size calculation are described in Supplementary material 2 (Statistical Analysis Plan).

### Recruitment and patient retention

Recruitment will be granted for every patient hospitalized with COVID-19, who will be screened for eligibility criteria, sign the Informed Consent Form and be followed by a local study team who have been properly trained until discharge. Loss to follow-up is not expected in this period. If hospital discharge occurs before 15 days after the randomization date, these patients will be evaluated by telephone call, which will be performed by the center 15 days after randomization and 28 days later. Those who return home before D10 will be assessed daily until completion of the study drug treatment period (10 days) by telephone call or in person on D3, 6 or 10 to collect nasal swabs and to assess treatment compliance.

### Sequence allocation

The randomization list will be generated electronically using appropriate software. Randomization will be performed in blocks and stratified by center. In Phase II Stage 1, the blocks will have 12 codes (positions), with each treatment group represented by three different codes and each PbO by a single code. In Phase II, Stage 2, the blocks will have six codes, the treatment groups will be represented by two different codes, and each PbO will be represented by a single code. Block sizes will be adapted if any arm is discontinued. In the third stage of the study, the blocks will have six codes, four codes representing the treatment group and 2 codes representing the PbO group.

### Allocation concealment

The concealment of the randomization list will be maintained through a centralized, automated, internetbased randomization system, available 24 hours a day, developed by a team of programmers and researchers from the Research Institute of *HCor-Hospital do Coração* (HCor).

### Blinding

This study is not a global double-blind study in Stages 1 and 2, as we have 3 drugs with different physical characteristics, rendering global blinding impossible. Both the participant and investigator can know, after randomization, to which drug group the patient was allocated. However, none will know whether the capsule or coated tablet to be administered contained the active drug or PbO, ensuring blinding of participants and investigators within that specific group, as well as the outcome assessors.

In Phase 3, the global blinding of the stage is possible, since we will have only one active group and a PbO.

A partnership will be established with a handling pharmacy duly licensed to carry out the fractioning, repacking, labeling and blinding process of the drugs. The pharmacy will deliver all the drugs to a logistic contractor, who will deliver them to the centers.

### Data collection, management and analysis

#### Collection and management methods

The data collection system to be adopted in the development of this study is widely used in research projects and easily accessible via the web with Redcap^®^ software. Only users qualified by the system administrator receive access, each registered user accesses the system only with their login and password, and the sharing of this information between project collaborators is prohibited.

The data collected and the participant timeline are described in table 5.

**Table 5:** Participant timeline and data collection

				Onset: April 2021							End: March 2022				
**Screening**	-t1	T1	D1	D2	D3	D4	D5	D6	D7	D8	D9	D10	D14	D15	D28
Screening	x														
Clinical history	x														
Eligibility	x														
ICF	x														
Screening tests	x														
ECG	x				x										
Allocation		x													
**Interventions**	**-t1**	**T1**	**D1**	**D2**	**D3**	**D4**	**D5**	**D6**	**D7**	**D8**	**D9**	**D10**	**D14**	**D15**	**D28**
Intervention drug/drugs			x	x	x	x	x	x	x	x	x	x			
Placebo			x	x	x	x	x	x	x	x	x	x			
**Data and tests collection**	**-t1**	**T1**	**D1**	**D2**	**D3**	**D4**	**D5**	**D6**	**D7**	**D8**	**D9**	**D10**	**D14**	**D15**	**D28**
Demographic data	x														
RT-PCR	x														
Safety checks	x				x			x				x			
Baseline data			x												
Concomitant therapies			x	x	x	x	x	x	x	x	x	x			
Daily data			x	x	x	x	x	x	x	x	x	x	x	x	x
Primary outcome		x			x			x				x		x	
Secondary outcomes			x	x	x	x	x	x	x	x	x	x	x	x	x

D - day; ICF - Informed Consent Form; ECG - electrocardiogram; RT-PCR - real-time polymerase chain reaction.

#### Retention

Data will be collected from patients admitted to the ICU/hospital, which reduces the risk of data loss due to loss of follow-up. Telephone contact will be made centrally by professionals who have not participated in other stages of the study.

- **Postdischarge follow-up:** Patients discharged less than 15 days after randomization will be contacted by telephone daily until D10 and on D15 and 28 and questioned using a structured form by an interviewer who is blinded to the research participant’s allocation group. In this contact, the participant or his or her family member will be asked about the observation of adverse events, and ordinal scales of 6 and 7 points will be applied for the secondary outcomes.

### Statistical methods

The main analysis of the data from this study will be performed on patients of the intention-to-treat (ITT) population. The definition of the analysis populations and more details on statistical methods are available in the Statistical Analysis Plan. The analyses will be performed with R software (R Core Team, 2020).

All the different PbOs for ATV, DCV and SOF will always be analyzed together as a single PbO group.

### Analysis of primary outcomes

#### Phase II/1

In Phase II/1, patients will be allocated in ratio of 3:3:3:1:1:1 (3 for each treatment group and 1 for PbO). For Phase II/1, the parameter of interest for the decision will be the comparison of the decay rates of the viral load logarithm from real time polymerase chain reaction (RTPCR) to COVID-19 in 9 days between the treatment groups compared with the control using a mixed linear model.

The interim analysis, which is planned to begin when 126 patients are randomized and followed for at least 10 days, will be exclusively conducted for a patient safety assessment (based on safety outcomes and adverse events).

At the end of Phase II/1, each group will be compared with the PbO group considering a significance level for each comparison of 0.067 (Bonferroni correction for multiple comparisons), such that the global type I error of Phase II/1 will be 0.20. If no treatment is significantly different from the PbO group at the stipulated level of significance, the study will be terminated at this stage.

Among the treatments significantly different from the PbO group in relation to the linear rate of viral load decay, the one with the highest rate of decay and considered safe by the independent study safety committee will be a candidate treatment for inclusion in Phase II/2 of the study. It will be combined with the drug responsible for the second highest linear decay rate to comprise the second Phase II/2 treatment group.

#### Phase II/2

In the 2^nd^ stage, the patients will be allocated in the ratio of 2:2:1:1, 2 for each active arm and 1 for each PbO, and the same statistic will be used as in Phase II/1. Details are described in the Statistical Analysis Plan - SAP (Supplementary material 2).

As in Phase II/1, the interim analysis planned after inclusion and data are collected from half of the planned patients will only assess safety data.

#### Phase III

The third stage of the study will proceed in a 2:1 allocation (two active treatments for each PbO), with a minimum inclusion of 189 patients and a maximum of 564. The study will perform interim analyses that may interrupt the study due to safety, futility, or efficacy, using all participants already randomized in Phases II/1 and II/2, since the primary efficacy outcome of Phase III (time of use of ventilatory support within 15 days) will be analyzed for all patients from these earlier phases as well.

The hypothesis test for the treatment effect will be performed using a generalized additive model of location and scale considering the distribution of a beta-binomial mixture with inflated zeros for the data adjusted by age and considering the random effect of the center for each intercept (model for the beta-binomial part and for the probability of zeros).

Phase III has three interim analyses planned, m = 3, for each third of the collected sample. The interim analyses consider stop limits according to the O’Brien-Fleming criterion, with significance levels of 0.06%, 1.51% and 4.71%, respectively, to maintain the global significance level at 5%. In Phase III, discontinuations due to futility will be allowed and use the same stopping criteria defined for efficacy.

Although the PbO group is composed of individuals receiving PbOs for three separate drugs (sometimes in combination), it will always be evaluated as a single group after gathering information from all “PbO arms”.

Secondary/exploratory outcomes and additional analyses are described in Supplementary material 1 (Section S4.1).

#### Interim analyses

One interim analysis is planned in Phase II (first stage), 1 in Phase II (second stage) and 3 in Phase III, and 3 interim analyses will be conducted, as described in detail in the statistical methods section.

The database will be blocked after obtaining a 15day follow-up for all patients, and all necessary actions to obtain follow-up data will be performed. All interim analyses would be made available to *Agência Nacional de Vigilância Sanitária* (Anvisa).

### Ethical aspects and good clinical practices

#### Ethical considerations and dissemination

The trial was designed according to the guidelines for good clinical practice, follows the principles of the Declaration of Helsinki, and was approved by the *Comissão Nacional de Ética em Pesquisa* (Conep; n° 4.799.171, June 23, 2021), Anvisa (n° 107/2020) and the Ethics Committee (EC) guidelines at each center.

Patients are included after the signed Informed Consent Form is obtained by the investigators participating in the study. In addition, all eventual amendments to the protocol must be approved by the REC/Conep system before its implementation by the participating centers.

The study will be submitted for publication after completion, regardless of its findings. Manuscript preparation will be an inalienable responsibility of the Steering Committee. The main paper will be authored by the Steering Committee members plus the principal investigators of the ten top recruiting sites, who will contribute intellectually to the manuscript.

### Adverse events

All adverse events will be recorded by the investigators on the clinical records (CRF), regardless of severity, and graded according to the Division of AIDS (DAIDS) Severity table. Table 1S (Supplementary material 1) shows the known adverse events resulting from the use of the products under investigation and explains the main actions to implement for SAEs.

The principal investigator at the research center is responsible for informing research ethics committees of any SAEs within 24 hours, as required by local regulations, except those that are classified as study outcomes.

The HCor Research Institute is responsible for receiving and monitoring all adverse events that occur during the clinical study. All adverse events classified as serious according to the definition of RDC Anvisa 09/15 must be informed to the sponsors within a maximum period of 24 hours of knowledge through a specific email. After evaluation, Fiocruz and Blanver Farmoquímica e Farmacêutica must notify Anvisa of serious, unexpected reports whose causality is possible, probable or defined in relation to the product under investigation within the regulatory deadlines.

### Study organization

#### Protocol violations and deviations

Major deviations related to the inclusion or exclusion criteria, study conduction, and patient management must be reported to the coordinating site.

#### Trial organization and oversight

The Steering Committee comprises the study investigators and is responsible for the development of the study protocol, manuscript drafts and study submission for publication. A team from the HCor Research Institute coordinates the study in association with the Brazilian Research in Intensive Care Network (BRICNet) and Coalition Brazil. The HCor Research Institute has been responsible for conducting the study since its inception and managing and controlling data quality. The Data Safety and Monitoring Board (DSMB) is composed of an external statistician and 2 other researchers who are experts in critical care medicine. The DSMB is responsible for the interim analysis and for providing guidance to the Steering Committee regarding the continuation and safety of the trial after the interim analyses.

#### Current status

We randomized 256 patients from April until July 2021. The first DSMB meeting occurred in May and June 2021. After analyzing data according to DSMB Charter, the DSMB recommended continuing the trial as planned.

### Dissemination policy

The results of the study will be disseminated in presentations at congresses and submitted for publication in high impact scientific journals.

## DISCUSSION

This manuscript describes the protocol of the REVOLUTIOn trial, which is a randomized, PbO-controlled, adaptive, multiarm, multistage study to evaluate the efficacy of ATV, DCV and SOF simultaneously compared with their PbOs. The main objective is to identify whether any of these drugs alone or in combination are capable of reducing viral load and, if so, investigate the clinical outcome of increasing the number of days free of respiratory support in a larger population.

Expeditious and methodologically rigid clinical trials are demanding to identify safe and effective treatments. Nevertheless, rapid identification and abandonment of harmful or ineffective approaches are equally important, especially during a pandemic. The principal aim of the REVOLUTIOn trial is to aid in the recognition of any of these 3 repurposed antivirals as treatments for COVID-19. The multiarm, multistage design of REVOLUTIOn helps rapidly identify the effectiveness of these drugs in a seamless manner and at the same time, starting with a Phase II clinical trial focusing on safety and ending with a Phase III trial capable of answering clinically important questions.

Repurposed drugs are important in resource-limited settings because the interventions are more rapidly available, have already been tested safely in other populations and are inexpensive.

Limitations of the protocol are described below. First, decisions related to changing stages rely on a decreasing viral load in the initial phases despite extensive debate on the association of viral load with worse clinical outcomes.^(12)^ Second, late initiation in recruiting participants in the pandemic timeline slowed our recruitment rate, as pandemic cases steadily decreased after vaccination outset and, third, late protocol publication, although the content of this protocol paper is absolutely consistent with the protocol submitted and approved by the national/local Ethics Research Committee, our national regulatory agency (Anvisa) and ClinicalTrials.gov.

## CONCLUSION

In conclusion, the REVOLUTIOn study protocol explains the design and purpose of the study, which has the objective of answering whether repurposed antiviral drugs are effective and safe treatments for COVID-19 in an adaptive, multiarm, multistage design starting with a Phase II clinical trial and ending with a Phase III trial.

## Figures and Tables

**Table:** Summary of versions

Version	Date
v4.0	Feb 26th / 2021
v3.0	Dec 28th / 2020
v2.0	Oct 30th / 2020
v2.0	Oct 15th / 2020
v1.0	Sep 09th / 2020
v1.0 August 2020	Aug 07th / 2020
Original project v1.0 July 2020	Jul 27th / 2020
